# Factors associated with utilization of long term family planning methods among women of reproductive age attending Bahir Dar health facilities, Northwest Ethiopia

**DOI:** 10.1186/s13104-018-4031-0

**Published:** 2018-12-27

**Authors:** Endalamaw Tesfa, Hailyesus Gedamu

**Affiliations:** 1 Department of Biochemistry, College of Medicine and Health Science, Bahir Dar University, P.O. Box 79, Bahir Dar, Ethiopia; 2 Department of Nursing, College of Medicine and Health Science, Bahir Dar University, P.O. Box 79, Bahir Dar, Ethiopia

**Keywords:** Long term family planning, Health facility, Reproductive age women, Bahir Dar

## Abstract

**Objective:**

This health institution based cross section study was designed to determine factors associated with utilization of long term family planning methods among women reproductive age attending Bahir Dar health facilities.

**Result:**

A total of 406 women were interviewed in this study. The mean age (standard deviation) of the study participants was 26.96 ± 6.31. About 99% of the study participants were consisted from Amhara ethnic group and 60.6% of them urban dwellers. In this study about 90.9% of the study participants had information about LTFP methods and 26.4% of them utilizing the methods. Factors like; knowledge of the women towards LTFP, spousal discussion on FP and occupation of the women affects LTFP utilization (6 times, 3 times and 4 times, respectively) when compared with their counter parts. In addition monthly income of the household was also associated to LTFP methods. In this study less percentage (26.4%) of women’s utilizing LTFP methods that were significantly associated with the knowledge of women on LTFP, spousal discussion on FP, occupation of the women and monthly income of the household. As result continuous health education will be recommended.

**Electronic supplementary material:**

The online version of this article (10.1186/s13104-018-4031-0) contains supplementary material, which is available to authorized users.

## Introduction

Continuous population growth was become an imperative problem for developing countries [1]. In sub-Saharan Africa like Ethiopia the population growth increases dramatically that adversely affect the socio economic development of the country. As a result, countries are enforced to develop population policy to limit population growth [2]. Family planning (FP) is a tool to control population growth [3]. FP is central to efforts to reduce poverty, promote economic growth, raise female productivity, lower fertility and improve child survival and maternal health. FP can prevent maternal deaths up to 20–35% [4]. Long term family planning (LTFP) methods had low failure rate, safer and cost effective than short acting contraceptives. They prevent pregnancy more than a year in one action without requirement of repeated procedures [5]. Despite its effectiveness, improve maternal health, reduce population growth and reversibility of fertility the acceptance and utilization of LTFP methods were very poor [6, 7].

In sub- Sahara Africa utilization LTFP method was very low [8]. According to the Ethiopian demographic health survey (EDHS) mini report in 2014 the prevalence of LTFP method was relatively low [9]. There are several factors that contribute for low prevalence LTFP methods; side effects of the methods, lack of access to the methods, lack of information on the methods, maternal education [10–12]. Monthly income of the household and residence are determinants of LTFP methods [13, 14]. This study was designed to assess the factors associated to utilization of LTFP methods among women reproductive age attending Bahir Dar health facilities, Northwest Ethiopia.

## Main text

### Methods

#### Study design, study population and sampling

Health institution based cross-sectional study was conducted at Bahir Dar town health facilities from May to June, 2017. The town had two governmental hospitals and ten health centers that provide FP services. All reproductive age (15–49) women users of FP method coming to Bahir Dar town health facilities were our source population. All reproductive age women obtaining FP methods or FP counseling service during the study period were our study population. Utilization of LTFP method was a dependent variable. Socio-demographic variables, obstetric variables and other clinical variables were considered as an independent variable. LTFP operationally defined as contraceptive methods that delay pregnancy for 1 year and above (implants and intrauterine contraceptive devices).

A simple random sampling technique was applied to get the study participants. The sample size was estimated by using single proportion formula. The total sample size was 406. The calculated sample was allocated into four governmental health facilities (Han, Shimbit, Zenzelma health centers and Addis Alem hospital) the detail sampling procedure attached as Additional file 1.

#### Data collection and analysis

Data was collected after we obtained informed verbal consent from each participant by using interviewer administered structured questionnaire. The questionnaire was prepared in English then translated into Amharic later retranslated into English. Four BSc midwives and two BSc nurses were selected for data collection and supervision, respectively. Training was given for data collectors and supervisors to maintain data quality. Before the actual data collection pre-test were conducted in Durebete Health Center. Based on the pretest result, questionnaires were revised. Data were analyzed by using SPSS version 20 software. Descriptive statistics, binary and multiple logistic regressions was computed. Those variables were significant at P-value ≤ 0.2 were entered into multivariate analysis. The odds ratio was calculated to assess the association and strength of association of variables. P-value < 0.05 was taken as a cut point.

#### Ethical consideration

Ethical clearance was obtained from Bahir Dar University, College of Medicine and Health Sciences ethical review committee. Permission was also requested from the administrators of four health institutions. We precede our data collection after we obtained verbal informed consent from each study participants.

### Results

#### Socio-demographic characteristics

A total of 406 individuals included in this study. The mean age (standard deviation) of the study participant was 26.96 ± 6.31. In this study almost all (99%) of the study participants were consisted from an Amhara ethnic group. Majority of the study participants were a follower of Orthodox Christian religion (85.5) and lived in the urban area (60.6%). Most of them were married (81.5%). The mean age of marriage and first delivery was 18.82 ± 2.45 and 20.86 ± 2.87, respectively (Table 1).

**Table 1:** Socio- demographic characteristics of women attending Bahir Dar health facilities, Northwest Ethiopia, 2017

Variables	Response	Frequency	Percentage
Age (years)	15–19	71	17.5
	20–24	145	35.7
	25–29	109	26.8
	≥ 30	81	20.0
Marital status	Single	56	13.8
	Married	331	81.5
	Divorced	4	1.0
	Widowed	15	3.7
Residences	Urban	246	60.6
	Rural	160	39.4
Religion	Orthodox Christian	348	85.7
	Muslim	53	13.1
	Other	5	1.2
Ethnicity	Amhara	402	99.0
	Other	4	1.0
Educational status	No formal education	111	27.3
	Primary education	149	36.7
	Secondary education	74	18.3
	College and university	72	17.7
Availability of health facility at a distances of 5 km	Yes	305	75.1
	No	101	24.9
Occupation	Government employed	76	18.7
	Housewife	144	35.5
	Merchant	90	22.2
	Student	56	13.8
	Other	40	9.8
Monthly income in Ethiopian Birr	< 1000	44	10.8
	1001–2000	50	12.3
	2001–3500	112	27.6
	3501–5000	72	17.7
	> 5000	128	31.5

#### Reproductive characteristics of LTFP utilization

Almost all (99.5%) of the study participants had knowledge about modern FP methods and 90.9% of the women had information about LTFP methods. About 64.5% of the study participants were pregnant of this 51.3% of them were become pregnant two and < two times. In this study the major reason of women not utilizing LTFP method were fear of side effect, lack of information and need of more children accounts 66.9%, 12.4% and 5.4%, respectively (Table 2).

**Table 2:** Reproductive characteristics of women attending Bahir Dar health facilities, Northwest Ethiopia, 2017

Variables	Response	Frequency	Percent
Knowledge on contraceptive	Yes	404	99.5
	No	2	0.5
Utilization of contraceptive	Yes	371	91.5
	No	35	8.6
Choice of methods	Injectable	225	55.4
	Implant	103	25.4
	Oral contraceptive	34	8.4
	Emergency	5	1.2
	IUCD	4	1.0
Knowledge on LTFP methods	Yes	369	90.9
	No	37	9.1
LTFP utilization	Yes	107	26.4
	No	299	73.6
History of pregnancy	Yes	261	64.5
	No	145	35.7
Gravidity	≤ 2 Pregnancies	134	51.3
	> 2 Pregnancies	127	48.7
Parity	≤ 2 Alive children	131	54.1
	> 2 Alive children	111	45.9
Desire of more children	Yes	210	51.7
	No	196	48.3
Purpose of FP utilization	For spacing	304	74.9
	For limiting	102	25.1
History of abortion	Yes	57	14.0
	No	349	86.0
Spousal discussion on FP methods	Yes	324	79.8
	No	82	20.2
Accessibility of FP methods	Yes	392	96.6
	No	14	3.4
Birth interval between children (if they have ≥ 2 children) (years)	≤ 2	5	2.7
	> 2	178	97.3
Reason not taking LTFP methods	Lack of information	37	12.4
	Fear of side effect	200	66.9
	Need of more children	16	5.4
	Other	46	15.4

*LTFP* long term family planning, *FP* family planning, *IUCD* intrauterine contraceptive device

#### Factors affecting LTFP utilization

Thirteen independent variables were analyzed in logistic regression to know their association. Variables which were significant at P ≤ 0.2 entered into multivariate logistic regressions. Out of thirteen variables four were significantly associated with LTFP methods. These are knowledge to LTFP, spousal discussion on FP methods, occupation and monthly income. Those participants who were merchants in occupation had 4 times more likely to use LTFP than others. Women who have knowledge on LTFP were about 6 times more likely to practice LTFP methods than women who don’t have knowledge. Women who discussed about LTFP methods with their partners had 3 times more likely to utilize LTFP than their counterparts (Table 3).

**Table 3:** Logistic regression analysis of women attending Bahir Dar health facilities, Northwest Ethiopia, 2017

Variables	Response	Utilization of LTFP		COR at 95% CI	Sig	AOR at 95% CI
		Yes	Total			
Age of the respondent (years)	15–19	12	71	2.070 (0.946, 4.528)	0.068	0.945 (0.401, 2.231)
	20–24	32	145	1.487 (0.802, 2.757)	0.208	0.526 (0.197, 1.405)
	25–29	39	109	0.756 (0.408, 1.401)	0.374	0.534 (0.178, 1.604)
	≥ 30	24	81	1	1	1
Marital status	Single	8	56	1	1	1
	Married	97	331	0.402 (0.183, 0.881)	0.023	0.394 (0.061, 2.556)
	Others	2	19	1.417 (0.273, 7.342)	0.678	0.415 (0.078, 2.208)
Residence	Urban	69	246	0.799 (0.505, 1.263)	0.337	1.581 (0.815, 3.067)
	Rural	38	160	1	1	1
Educational status	No formal educated	21	111	1	1	1
	Primary education	43	149	0.789 (0.400, 1.557)	0.494	0.787 (0.263, 2.357)
	Secondary education	14	74	1.000 (0.472, 2.119)	1.000	1.188 (0.422, 3.339)
	College and university	29	72	0.346 (0.177, 0.675)	0.002	2.245 (0.832, 6.058)
Occupation	Government employed	33	81	0.301 (0.112, 0.805)	0.017	1.798 (0.678, 4.770)
	Merchant	23	89	0.594 (0.219, 1.612)	0.306**	3.873 (1.155, 12.986)
	Student	6	57	1.759 (0.519, 5.956)	0.364	1.509 (0.552, 4.122)
	Housewife	39	144	0.557 (0.215, 1.444)	0.229	1.772 (0.460, 6.823)
	Other	6	35	1	1	1
Availability of health facility at 5 km	Yes	83	305	0.834 (0.494, 1.406)	0.495	0.988 (0.425, 2.296)
	No	24	101	1	1	1
Monthly income in Ethiopian Birr	< 1000	6	44	1.855 (0.714, 4.823)	0.205**	0.319 (0.104, 0.977)
	1001–2000	20	50	0.439 (0.218, 0.886)	0.021	0.754 (0.249, 2.284)
	2001–3500	29	112	0.838 (0.464, 1.515)	0.559	0.676 (0.208, 2.201)
	3501–5000	23	72	0.624 (0.327, 1.190)	0.152	1.047 (0.337, 3.249)
	> 5000	29	128	1	1	1
Knowledge of LTFP Methods	Yes	105	369	0.144 (0.034, 0.608)	0.008**	6.250 (1.326, 29.472)
	No	2	37	1	1	1
Spousal discussion on FP	Yes	98	324	3.517 (1.692, 7.312)	0.001**	2.398 (1.021, 5.633)
	No	9	82	1	1	1
Desire of more children	Yes	62	210	1	1	1
	No	45	196	1.406 (0.900, 2.195)	0.134	0.937 (0.539, 1.627)
History of pregnancy	Yes	81	261	0.486 (0.295, 0.800)	0.005	1.294 (0.607, 2.758)
	No	26	145	1	1	1
History of abortion	Yes	14	57	1.116 (0.584, 2.133)	0.740	0.896 (0.416, 1.932)
	No	93	349	1	1	1
Accessibility of FP methods	Yes	103	392	1.122 (0.344, 3.657)	0.848	0.344 (0.076, 1.549)
	No	4	14	1	1	1

** Shows statistical significant association in the adjusted odds ratio

### Discussion

In this study the overall utilization of LTFP methods among reproductive age women was 26.4%. The finding of this research was almost similar in studies conducted in Mbarara district and Areka town [11, 15]. The prevalence of this research result was slightly higher than in studies conducted in different parts of Ethiopia [16–21]. This higher prevalence might be due to the accessibility of health facilities, increased awareness of the community due to health extension workers and the study design.

In this study almost all 99. 5% of study participant have information about modern contraceptive methods and 90.9% of the study participant had information on LTFP methods. This is in line with 2014 Ethiopian Demographic Health survey mini report (96.5%). The prevalence of mothers that use any modern contraceptive methods and LTFP methods in this study was 91.4% and 26.4%, respectively [9]. This result lower than studies conducted in Kampala and Ethiopia [7, 12, 17, 22]. In this study factors like; knowledge of women towards LTFP method, habit of partner discussion, less than 1000 Ethiopian birr monthly income and become merchant by occupation of the women were found to be determinants of LTFP methods.

This study revealed that women who discussed with their husband about LTFP methods were three times more likely to use LTFP methods than their counter parts. This is supported by studies conducted in Uganda, Rwanda and Ethiopia [1, 4, 8, 16, 20, 22, 23]. Out of the variables which showed significant associations at the multi-variable logistic regression analysis, high odds of using LTFP methods were seen among women with knowledge of LTFP methods. This finding suggests that women with knowledge of LTFP methods are more likely to practice FP services than their counter parts. This finding was strengthened by other studies conducted in Ethiopia [1, 2, 7, 19, 23, 24]. In this study, merchants by occupation more likely to utilized LTFP methods than their counter parts. Occupation of the women was associated to FP utilization in different studies conducted in Ethiopia [15, 25–27]. Monthly income of the household was positively associated with LTFP utilization. However, after adjustment it doesn’t show significant association. This is supported by studies conducted in Ethiopia [21].

In this study knowledge of LTFP was relatively high (90.9%). However, its utilization was low (26.4%). This is due to factors like; knowledge of women to LTFP methods, habit of partner discussion, monthly income of the household and becoming merchant by occupation were found to be determinants of LTFP utilization. As a result, improving the norms of partner discussion and continuous health education will be encouraged. In addition to explore factors in detail another longitudinal study will be recommend.

## Limitations of the study

This study isn’t free from limitation. Its limitation relies on the method part; health institution based cross sectional study doesn’t much explore the determinants about LTFP methods like community based and longitudinal studies. As a result another longitudinal study will be necessary to explore determinants in detail. Numbers related to knowledge of FP methods might be relatively higher to give an inference to general population.

## Additional file

**Additional file 1: Figure S1.** Schematic presentation on sampling procedure of factors associated with utilization of long term family planning methods among women of reproductive age attending Bahir Dar health facilities, Northwest Ethiopia, 2017. **Figure S2.** Bar graph of prevalence of family planning utilization among reproductive age women in Bahir Dar town, Northwest Ethiopia, 2017.

## Abbreviations

FP: family planning
LTFP: long term family planning
IUCD: intrauterine contraceptive devices
CI: confidence interval
COR: crude odds ratio
AOR: adjusted odds ratio
HC: health center
