# Supplementary material for: Factors associated with utilization of long term family planning methods among women of reproductive age attending Bahir Dar health facilities, Northwest Ethiopia
Source: BMC Res Notes. 2018 Dec 27;11:926. doi: 10.1186/s13104-018-4031-0 (PMC6307261; doi:10.1186/s13104-018-4031-0)
Supplement: Supplementary file 1 — Additional file 1: Figure S1. Schematic presentation on sampling procedure of factors associated with utilization of long term family planning methods among women of reproductive age attending Bahir Dar health facilities, Northwest Ethiopia, 2017. Figure S2. Bar graph of prevalence of family planning utilization among reproductive age women in Bahir Dar town, Northwest Ethiopia, 2017. [file 13104_2018_4031_MOESM1_ESM.docx]

**Fig. S1** Schematic presentation on sampling procedure of factors associated with utilization of long term family planning methods among women of reproductive age attending Bahir Dar health facilities, Northwest Ethiopia, 2017.

**Figure S2.** Bar graph of prevalence of family planning utilization among reproductive age women in Bahir Dar town, Northwest Ethiopia, 2017.
